# P-type Inversion at the Surface of β-Ga_2_O_3_ Epitaxial Layer Modified with Au Nanoparticles

**DOI:** 10.3390/s22030932

**Published:** 2022-01-25

**Authors:** Maciej Krawczyk, Ryszard Korbutowicz, Rafał Szukiewicz, Patrycja Suchorska-Woźniak, Maciej Kuchowicz, Helena Teterycz

**Affiliations:** 1 Faculty of Electronics, Photonics and Microsystems, Wrocław University of Science and Technology, Wybrzeże Wyspiańskiego 27, 50-370 Wroclaw, Poland; ryszard.korbutowicz@pwr.edu.pl (R.K.); patrycja.suchorska-wozniak@pwr.edu.pl (P.S.-W.); helena.teterycz@pwr.edu.pl (H.T.); 2 Institute of Experimental Physics, University of Wrocław, M. Borna 9, 50-204 Wroclaw, Poland; rafal.szukiewicz@uwr.edu.pl (R.S.); maciej.kuchowicz@uwr.edu.pl (M.K.)

**Keywords:** semiconducting metal oxide, gas sensors, thin film

## Abstract

The electric properties and chemical and thermal stability of gallium oxide β-Ga_2_O_3_ make it a promising material for a wide variety of electronic devices, including chemiresistive gas sensors. However, p-type doping of β-Ga_2_O_3_ still remains a challenge. A β-Ga_2_O_3_ epitaxial layer with a highly developed surface was synthesized on gold electrodes on a Al_2_O_3_ substrate via a Halide Vapor Phase Epitaxy (HVPE) method. The epitaxial layer was impregnated with an aqueous colloidal solution of gold nanoparticles with an average diameter of Au nanoparticle less than 5 nm. Electrical impedance of the layer was measured before and after modification with the Au nanoparticles in an ambient atmosphere, in dry nitrogen, and in air containing dimethyl sulfide C_2_H_6_S (DMS). After the impregnation of the β-Ga_2_O_3_ epitaxial layer with Au nanoparticles, its conductance increased, and its electric response to air containing DMS had been inversed. The introduction of Au nanoparticles at the surface of the metal oxide was responsible for the formation of an internal depleted region and p-type conductivity at the surface.

## 1. Introduction

The properties of gallium oxide β-Ga_2_O_3_, such as ultrawide bandgap, high breakdown voltage, high carrier mobility, and chemical and thermal stability; and the fact that high quality bulk crystals (and therefore also monocrystalline substrates) can be fabricated with melt-growth methods [1,2] predestine β-Ga_2_O_3_ to be the material of choice for high power and high frequency electronic devices [3], solar-blind photodetectors [4,5], UV emitters, transparent conductive films, and gas sensors [6,7]. However, due to its electric properties, p-type doping of β-Ga_2_O_3_ still remains a challenge [2]. Many reports of highly oriented and high quality β-Ga_2_O_3_ thin films can be found in the literature [8]. The synthesis is carried out by methods such as Halide Vapor Phase Epitaxy (HVPE) [9], Chemical Vapor Deposition (CVD), Metalorganic Vapor Deposition (MOCVD) [10], Molecular-Beam Epitaxy (MBE) [3], Pulsed Laser Deposition (PLD) [5], magnetron sputtering [4], evaporation in oxygen plasma [11], and sol-gel [12]. In the vast majority of these methods, the synthesis is carried out at reduced pressure, or in a vacuum; however, regardless of the conditions of synthesis, their common goal is to produce smooth, homogenous layers for later applications in semiconductor devices. On the other hand, for gas sensor applications, layers with highly developed surfaces are desirable. The larger the specific surface of the gas sensitive material, the greater the concentration of active centers that determine the sensitivity of the sensor material. It is widely believed that materials with large active surface areas are characterized by very good sensitivity [13,14].

Stoichiometric, undoped β-Ga_2_O_3_ is an isolator; however, crystals grown at low partial pressure of oxygen exhibit n-type conductivity at high temperature. Opinions on the source of n-type conductivity are divided. The literature attributes it to unintentional dopants (introduced during the process of synthesis) as well as the presence of various types of defects in the crystal structure of this material: oxygen vacancies, hydrogen interstitials and substitutions, and Ga interstitials [2,15,16]. Experimental studies show that the resistivity of the crystalline grains’ boundaries, but also their bulk, increases with the concentration of oxygen in the ambient atmosphere [17]. This relationship is explained by the adsorption and desorption of oxygen ions at the surface of the metal oxide, and by the diffusion of oxygen atoms from the ambient atmosphere to the crystal lattice, which causes a decrease in the number of oxygen vacancies. The ions adsorbed at the surface of the metal oxide can react with the reducing gases present in the atmosphere. As a result of these reactions, electrons bound at the surface of the oxide are released to the bulk of the material, which narrows the depleted layer at the grains’ boundaries and increases the conductivity of the material.

The literature reports on chemiresistive oxygen concentration sensors based on undoped β-Ga_2_O_3_ thin films operating at high temperature (1000 °C) [18,19] and, more recently, on β-Ga_2_O_3_ thin films doped with Si, and Cr_2_O_3_ operating at a medium temperature (400 °C) [20,21]. The conductivity of thin and thick films of gallium oxide increases in the presence of ethanol, methanol, carbon monoxide, acetone, hydrogen, hydrogen sulfide, and ammonia in the ambient atmosphere [22,23,24]. Moreover, β-Ga_2_O_3_ thin films exhibit hydrophobic properties, which is why the humidity of the ambient atmosphere has little effect on their gas sensing properties, even at room temperature [24]. It is worth noting that the majority of the thin film β-Ga_2_O_3_ gas sensors presented in the literature use electrodes made of platinum. Given the intended high temperature of operation, the use of platinum is justified; however, it is often overlooked that the Pt/β-Ga_2_O_3_ interface can behave like a Schottky diode and that it can affect the detection mechanism and even dominate it. The reason for this is a significant difference between the work functions of β-Ga_2_O_3_ (4.1 eV) [25] and Pt (5.65 eV) [26].

Gold nanoparticles are commonly used as a growth catalyst of β-Ga_2_O_3_ fibrous nanostructures (e.g., nanowires), where they remain anchored at the top of the growing structures [27,28,29,30]. However, Lu et al. [31] have researched the photocatalytic activity of β-Ga_2_O_3_ nanowires surface-decorated with 4–8 nm Au nanoparticles with a distribution density of approximately 420/µm^2^. The comparison of dark current and UV photocurrent of pristine and Au-decorated β-Ga_2_O_3_ nanowires has shown that the dark current decreases after modification with gold, while the UV photocurrent increases. The literature reports that β-Ga_2_O_3_ thin films modified with Au nanoparticles are scarce. Zhang et al. [32] have deposited β-Ga_2_O_3_ thin films on thin films made of gold nanoparticles. Similarly, they have observed an increase of UV photocurrent density for layers deposited on Au nanoparticles. Zhang et al. have explained this by the influence of Au nanoparticles acting as electron sinks that inhibit the recombination of electron-hole pairs under the UV light. To the best of our knowledge, the only gas sensor based on β-Ga_2_O_3_ thin film modified with Au particles described in the literature is to be found in the work of Schwebel et al. [33], who prepared a CO gas sensor. Au particles ranging in size from 10 nm up to 300 nm were deposited on the surface of β-Ga_2_O_3_ thin film by a wet chemical method. The resistance of the thin film increased after the modification with Au.

Currently, research on gas sensors is carried out in two main directions [34,35]. One is aimed at searching for gas-sensitive materials, including nanomaterials [14], with improved gas sensing parameters. The other is aimed at understanding the phenomena occurring during the operation of sensors, because this would facilitate the development of sensors with better usable parameters [36]—this aspect is the focus of this paper.

This article presents research on a β-Ga_2_O_3_ epitaxial layer impregnated with gold nanoparticles and the influence of this modification on the inversion of the type of electric conductivity at the surface of the layer. A β-Ga_2_O_3_ layer with an average thickness of 2.2 µm was synthesized on gold interdigitated electrodes on an Al_2_O_3_ substrate by the Halide Vapor Phase Epitaxy method. The study of the microstructure, the crystal structure, and the chemical composition of the obtained layer is presented. The impedance of the epitaxial layer in the atmosphere of ambient air, dry nitrogen, and air containing dimethyl sulfide (DMS) was investigated with impedance spectroscopy method. After the modification with Au nanoparticles, the conductance of the β-Ga_2_O_3_ epitaxial layer increased and its electric response in the atmosphere of dimethyl sulfide was inversed to p-type.

## 2. Materials and Methods

Interdigitated electrodes (IDE), leads, and soldering pads were screen printed with 8846-G gold paste (ESL Europe, Reading, UK) on the top surface of alumina substrate (96% Al_2_O_3_, CeramTec, Plochingen, Germany) with dimensions 25.4 mm × 2.5 mm × 0.254 mm. A meander-shaped heater was screen printed on the bottom surface with 5545 platinum paste (ESL Europe, Reading, UK). The heater leads and soldering pads were made with gold paste. The heater was covered with dielectric thick film (ESL Europe, Reading, UK). The substrate was fired for 10 min at 850 °C in the atmosphere of air. Before the synthesis of the epitaxial layer, the substrate was washed in an ultrasonic bath, first with isopropanol, and then with deionized water. Finally, the substrate was left to dry in the ambient atmosphere.

The synthesis of the β-Ga_2_O_3_ layer by Halide Vapor Phase Epitaxy method was carried out in a quartz tube reactor placed in a resistance furnace. The carrier gas was nitrogen obtained from liquefied nitrogen (Linde, Krakow, Poland) purified by GateKeeper inert gas purifier (Entegris, Billerica, MA, USA). The source of gallium was liquid metallic 6N purity grade gallium Ga (ITME, Warsaw, Poland). Gaseous 5.5 purity grade hydrogen chloride HCl (Messer, Chorzow, Poland) was used to produce GaCl gallium monochloride, which is a volatile compound at high temperature. Synthetic air (5.5 purity 20.5 ± 0.5% O_2_ in 6.0 purity N_2_) was used as a source gas (Messer, Chorzow, Poland) to produce gallium oxide Ga_2_O_3_ in a reaction with GaCl.

The three-zone resistance furnace was set to 840 °C in the deposition zone. The temperature shelf in the chlorination zone was set to 860 °C. The crystallization temperature of gallium oxide β-Ga_2_O_3_ by HVPE method is typically higher than 1000 °C—then the growth rate is significant and the surface morphology of the epitaxial layer is suitable for semiconductor devices (smooth and mirror-like) [9,37]. The β-Ga_2_O_3_ epitaxial layer obtained at a lower temperature, i.e., 840 °C, was characterized by a highly developed surface, suitable for gas sensors [14]. The synthesis was carried out at atmospheric pressure. Nitrogen was flowing through the open quartz reactor as the main carrier gas at a rate of 6000 mL/min. The flow rate of the synthetic air was 1000 mL/min. A quartz boat with metallic gallium was placed in the high-temperature zone. Hydrogen chloride HCl was flowing through the boat at a rate of 30 mL/min along a diluting stream of 250 mL/min of nitrogen gas. A quartz stand with the alumina substrate was inserted into the heated furnace and placed in the deposition zone (840 °C). The entire substrate was masked, except the gold interdigitated electrodes, and gallium oxide was deposited for 50 min only in the area of IDE. There was no need to use bed layers that crystallize at a lower temperature because then the α-Ga_2_O_3_ would be obtained.

**Figure 1:**
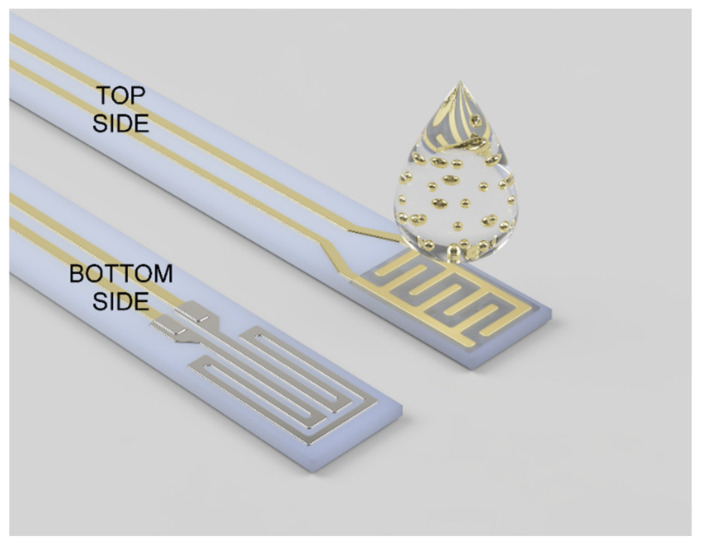
A render of the top and bottom layer of the ceramic substrate. The β-Ga_2_O_3_ epitaxial layer grown on the gold interdigitated electrodes was later impregnated with an aqueous colloidal solution of Au nanoparticles. The platinum heater made on the bottom of the substrate was covered with a thick dielectric film (not shown in the figure).

After characterization, the obtained gallium oxide layer was impregnated with 25 µL of an aqueous colloidal solution of gold nanoparticles with a concentration of 200 ppm and an average diameter of Au particles of less than 5 nm [38] (Figure 1). The epitaxial layer was re-examined after modification with the Au nanoparticles. 

The thickness of the epitaxial layer was assessed using the Talysurf CCI (Taylor Hobson, Leicester, UK) interference optical profilometer. 

The microstructure was observed using the SEM SU6600 Scanning Electron Microscope (Hitachi, Hitachinaka, Japan) at an acceleration voltage of 15 kV. The distribution of gold particles on the surface of the epitaxial layer and its chemical composition after impregnation with Au nanoparticles were investigated using the Energy-Dispersive X-ray Spectroscopy EDS NORAN System 7 (Thermo Fisher Scientific, Waltham, MA, USA) built into the chamber of the Hitachi SU6600 SEM.

The crystal structure of the β-Ga_2_O_3_ epitaxial layer was investigated by X-ray Diffraction using the Empyrean diffractometer (Malvern Panalytical, Malvern, UK) equipped with the PIXcel3D detector. CuKα radiation with a wavelength of 0.15406 nm, a voltage of 40 kV, and a current of 40 mA were used. The test sample was placed on an antireflective Si backing. Bragg–Bentano geometry was used. The characteristic peaks on the X-ray diffractogram were fitted with the Voigt profile.

The chemical composition of the surface of the studied epitaxial layer was investigated with X-ray Photoelectron Spectroscopy (XPS). The non-monochromatized X-ray source based on Mg anode lamp (Mg Kα emission line) was used. High-resolution photoelectron energy spectra were recorded with AES/XPS system (Leybold–Heraeus, Cologne, Germany). Base pressure in the UHV chamber during measurements was lower than 1 × 10^−9^ mbar. All of the acquired spectra were calibrated to adventitious carbon C1s at 285 eV. The overall resolution of the spectrometer during measurements was 0.96 eV as a full width of half maximum (FWHM) of the Ag3d5/2 line. CasaXPS software (Casa Software, Teignmouth, UK) was used for XPS data deconvolution and surface composition analysis. 

The electrical characterization of the epitaxial layer of β-Ga_2_O_3_ was performed by impedance spectroscopy using the 1260 impedance analyzer (Solartron Analytical, Farnborough, UK). The impedance of the layer was measured in a frequency range of 1 MHz to 1 Hz with 2 V_RMS_ sinusoidal voltage. The measurements were made at the temperature of 600, 650, 700, and 750 °C. The impedance of the epitaxial layer was measured before and after its modification with gold nanoparticles, in ambient air (T_air_ ≈ 20 °C, RH_air_ ≈ 35%), dry nitrogen, and in ambient air containing 1–16 ppm of dimethyl sulfide (DMS). The sample was heated by the platinum meander heater made on the bottom surface of the substrate (Figure 1). The heater was powered by DC voltage generated by the E3632A power supply (Agilent Technologies, Santa Clara, CA, USA). The impedance analyzer 1260 was controlled by ZPlot software version 3.5f (Scibner Associates, Southern Pines, NC, USA). The electric equivalent model was fitted with ZView software version 4.0c (Scribner Associates, Southern Pines, NC, USA).

## 3. Results

### 3.1. Microstructure

**Figure 2:**
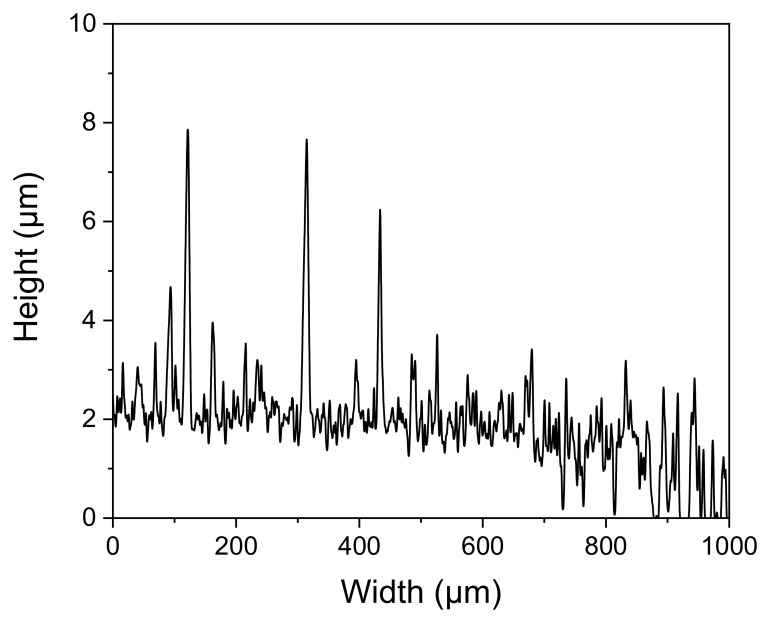
Profile of the surface of the β-Ga_2_O_3_ layer grown on the Si/SiO_2_ substrate.

The thickness of the obtained β-Ga_2_O_3_ layer was assessed based on a layer grown on a Si/SiO_2_ substrate. The synthesis was carried out under the same condition as for the layer grown on the alumina substrate. The average thickness of the layer was found to be approximately 2.2 µm. Additionally, the surface of the layer is highly developed (Figure 2).

**Figure 3:**
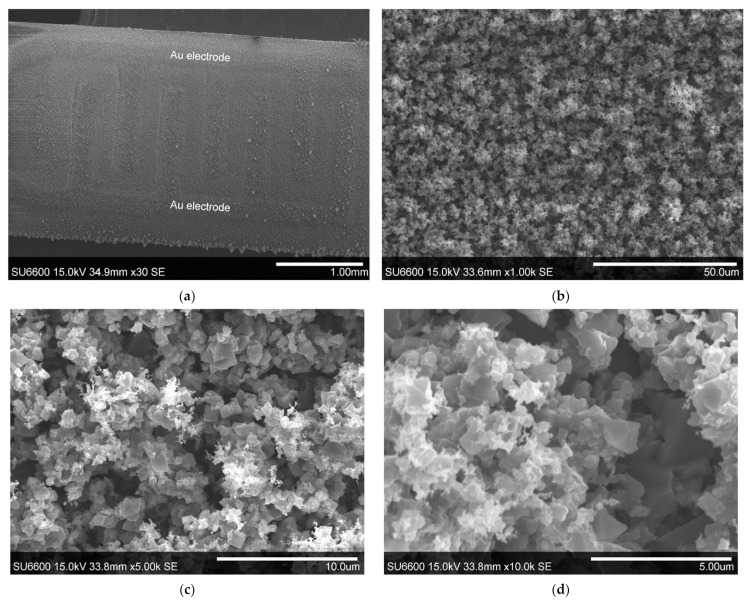
Microstructure of the surface of gallium oxide epitaxial layer: (**a**) under ×30 magnification. Gold interdigitated electrodes are visible under the epitaxial layer; (**b**) under ×1000 magnification; (**c**) under ×5000 magnification; (**d**) under ×10,000 magnification.

An accurate assessment of the surface microstructure of the layer grown on the alumina substrate was made using the scanning electron microscope. The observations have shown that the layer evenly covers both the substrate and the gold interdigitated electrodes (Figure 3a). The position of the electrodes is noticeable because their thickness is 12 ± 1 µm, which is about six times the thickness of the gallium oxide layer. The surface of the layer is highly developed and porous. It consists of grains of various shapes, with the majority of grains being clearly crystalline, as evidenced by the smooth sides of the grains (Figure 3). The average size of the crystalline grains is less than 1 µm.

Observations of the layer surface made after its impregnation with aqueous solution of Au nanoparticles did not reveal the presence of these nanoparticles, which is understandable given their very small diameter of less than 5 nm on average [38]. No changes in the microstructure of the layer itself were observed.

### 3.2. Crystal Structure

**Figure 4:**
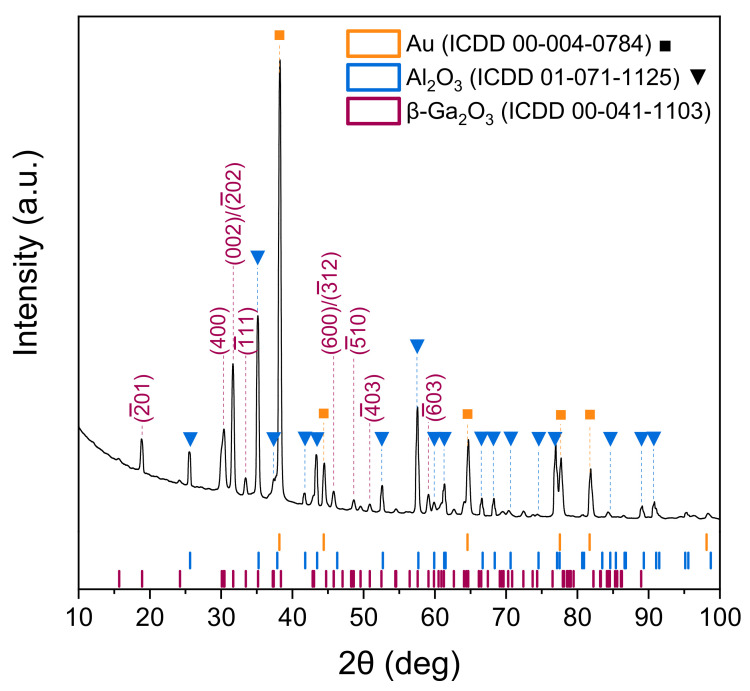
X-ray diffractogram of β-Ga_2_O_3_ epitaxial layer.

The analysis of the X-ray diffraction was conducted for the β-Ga_2_O_3_ epitaxial layer before it was modified with Au nanoparticles. The X-ray diffractogram was found to correspond with the standard data for β-Ga_2_O_3_ bulk monocrystal (ICDD 00-041-1103) with a monoclinic structure and belonging to the space group C2/m (Figure 4). The peaks corresponding to the studied epitaxial layer are slightly shifted relative to the standard data by less than −0.02°. The X-ray diffractogram also shows characteristic peaks from gold electrodes, as well as from the Al_2_O_3_ substrate. All of these peaks form Kα doublets [39], which, in some cases, overlap with peak characteristic to β-Ga_2_O_3_.

**Figure 5:**
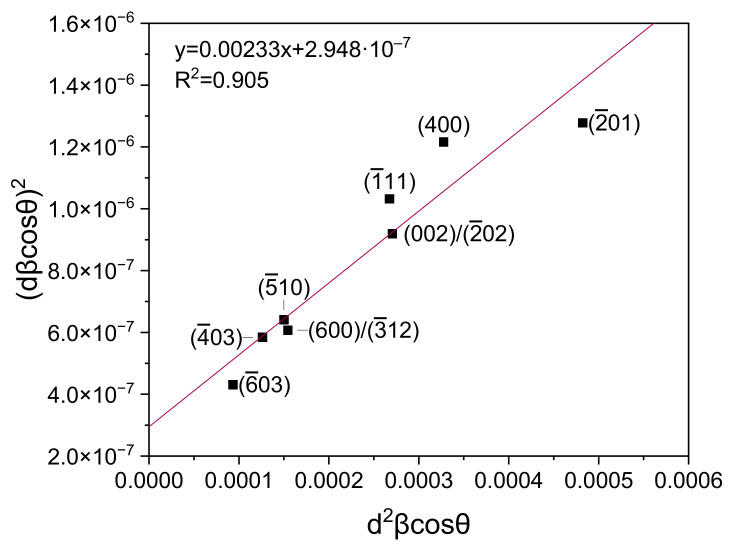
Size-Strain plot.

Due to the overlapping of peaks from different materials, peaks with 2*θ* angles of 18.88°, 30.06°, 31.66°, 33.43°, 45.79°, 48.62°, 50.85°, and 59.09° were used to calculate the strain and average crystallite size. The peaks correspond to the following crystallographic planes: ($\bar{2}$01), (400), (002)/($\bar{2}$02), ($\bar{1}$11), (600)/($\bar{3}$12), ($\bar{5}$10), (403), ($\bar{6}$03) (Figure 4). The average crystallite size *D* was determined from the slope of the Equation (1) plotted on the Size-Strain plot (Figure 5), and the strain *ε* was determined from the y-intercept [40,41]:

(1) $${\left(d\beta \cos\theta \right)}^{2}=\frac{k\lambda }{D}\left(d^{2}\beta \cos\theta \right)+\frac{\varepsilon^{2}}{4}$$

(2) $$D=\frac{k\lambda }{a}=59.5\;\mathrm{nm}$$

(3) $$\varepsilon =2\sqrt{b}=1.086\cdot 10^{-3},$$

where: *d*—interplanar spacing of the monoclinic system, *β*—broadening of the diffraction peak measured at half maximum (FWHM), *θ*—the scattering angle, *k* = 0.9—the shape factor for a spherical crystallite, *λ* = 0.15406 nm—X-ray wavelength, *D*—the average crystallite size, *ε*—the strain, *a*—the slope of the Equation (1), and *b*—y-intercept of Equation (1) (Figure 5).

By solving Equation (4) for the known values of Miller’s indices *h*, *k*, *l,* and sin^2^*θ* corresponding to characteristic peaks of the β-Ga_2_O_3_ epitaxial layer on the X-ray diffractogram, the parameters of the monoclinic unit cell *a*, *b*, *c*, *β*

(4) $$\sin^{2}\theta =\frac{\lambda^{2}}{4\sin^{2}\beta }\left(\frac{h^{2}}{a^{2}}+\frac{k^{2}\sin^{2}\beta }{b^{2}}+\frac{\ell^{2}}{c^{2}}-\frac{2h\ell \cos\beta }{ac}\right),$$

were calculated to be: *a* = 12.23 Å, *b* = 3.04 Å, *c* = 5.81 Å, *β* = 103.7°. The derivation of Equation (4) is given in [30]. The calculated parameters of the unit cell of β-Ga_2_O_3_ are very similar to the values given in the ICDD card 00-041-1103, which is understandable given that the 2*θ* angles of the characteristic peaks visible in the X-ray diffractogram, and therefore also the values of sin^2^*θ*, vary only slightly from the standard values.

### 3.3. Chemical Composition of the Surface

**Figure 6:**
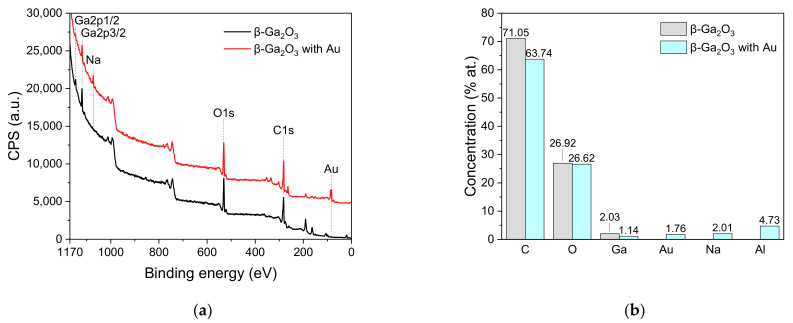
(**a**) XPS survey spectra of β-Ga_2_O_3_ epitaxial layer before and after impregnation with Au nanoparticles; (**b**) concentration of atoms present on the analyzed surfaces.

The surface composition of the β-Ga_2_O_3_ epitaxial layer before and after its modification with Au nanoparticles has been determined by X-ray Photoelectron Spectroscopy (XPS). Figure 6 presents the survey spectra of both analyzed surfaces. Sodium was found on the surface of the layer modified with Au nanoparticles, most likely due to the fact that Na was used as an acidity regulator during the synthesis of gold nanoparticles. Carbon C1s region mainly contains C-C, C-O, and carbonates bonds. The origin of these species is likely to stem from environmental contamination.

**Figure 7:**
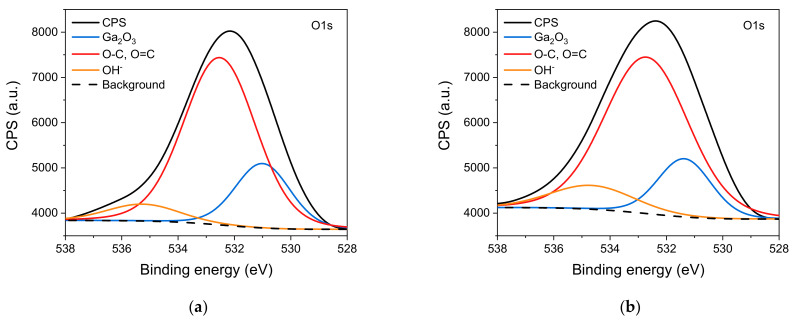
XPS spectra in the O1s region of: (**a**) unmodified β-Ga_2_O_3_ epitaxial layer; (**b**) β-Ga_2_O_3_ epitaxial layer modified with Au nanoparticles.

Ga_2_O_3_ network bonds and O-C and O=C bonds were observed in the O1s region (Figure 7). Additionally, a small amount of OH group was observed. Ga_2_O_3_ crystal lattice signal was more intense for the unmodified β-Ga_2_O_3_ epitaxial layer. The percentage of OH group bonds increased after modification with Au nanoparticles (Table 1).

**Figure 8:**
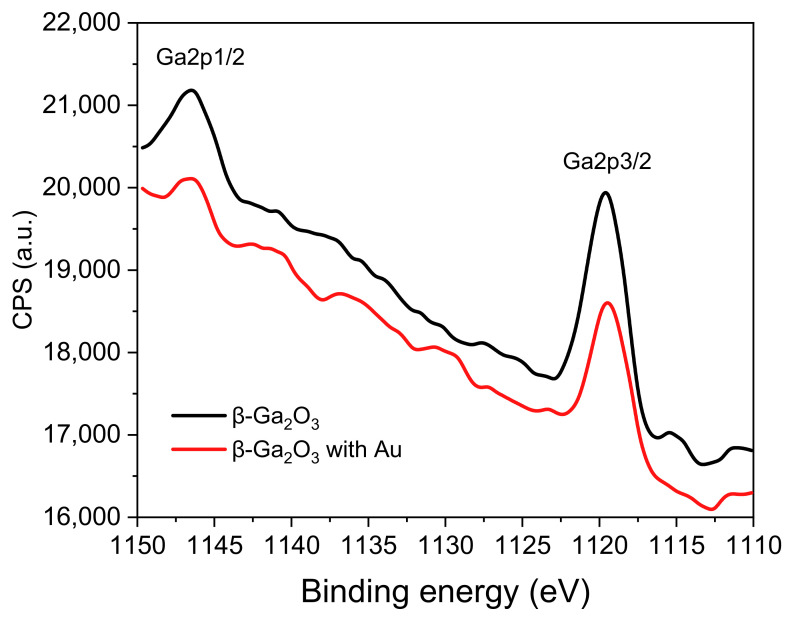
XPS spectra in the Ga2p region of β-Ga_2_O_3_ epitaxial layer before and after impregnation with Au nanoparticles.

The analysis of the Ga2p region has shown that Ga_2_O_3_ oxide was present at the surface of both of the studied epitaxial layers (Figure 8) [30].

### 3.4. Impedance Spectroscopy

**Figure 9:**
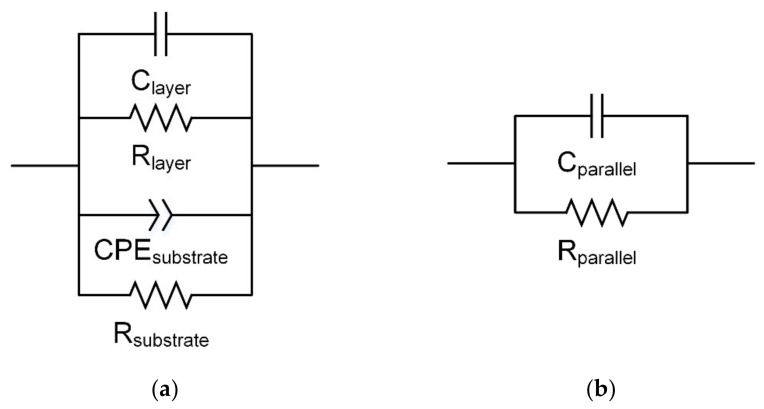
Electric equivalent circuits: (**a**) model of β-Ga_2_O_3_ epitaxial layer grown on Au interdigitated electrodes on Al_2_O_3_ substrate; (**b**) parallel RC connection used to analyze changes of the capacitance of the measured system in the frequency range below 10 Hz.

Gold interdigitated electrodes were printed on a separate Al_2_O_3_ ceramic substrate and their impedance in the ambient air atmosphere was measured. The measured impedance spectra were in the form of a single depressed semicircle; therefore, an electric equivalent model consisting of a parallel connection of a resistor *R*_substrate_ and a constant phase element *CPE*_susbtrate_ was proposed (Figure 9a). It was observed that in the measured range of 600 °C to 750 °C, the amplitude and the coefficient *α* of the constant phase element did not change and were, successively, 1.5 × 10^−10^ sΩ^−1^ and 0.954, while the value of *R*_substrate_ decreased with an increase in the temperature from 2.08 × 10^8^ Ω to 2.07 × 10^7^ Ω. 

The impedance spectra of the β-Ga_2_O_3_ epitaxial layer measured in the 1 MHz–1 Hz range are represented by single semicircles, which makes it difficult to extract the contributions to the impedance coming from the bulk of the β-Ga_2_O_3_ crystalline grains and their boundaries. Therefore, an electric equivalent circuit of the epitaxial layer consisting of a parallel connection of a resistor R_layer_ and capacitor *C*_layer_ was proposed (Figure 9a). The arrangement of the elements of this model were independent of the temperature, composition of the gas atmosphere, or the presence of gold nanoparticles at the surface of the layer; however, it should be noted that this circuit is not suitable for modeling the measured impedance spectra below 10 Hz. In addition, the capacitance of the epitaxial layer *C*_layer_ fitted in the 1 MHz–10 Hz frequency range was very small, in the order of 1 × 10^−20^ F. Changes in the capacitance of the measured system in the frequency range below 10 Hz were analyzed using a parallel RC circuit (Figure 9b). 

**Figure 10:**
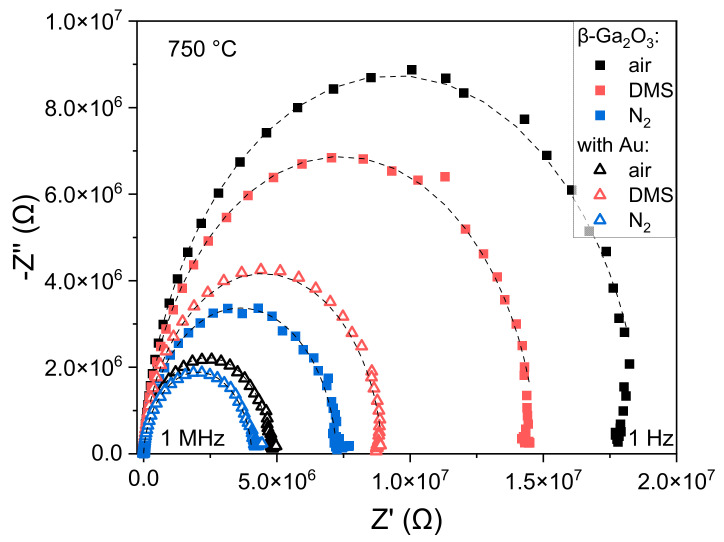
Impedance spectra measured at 750 °C of the β-Ga_2_O_3_ epitaxial layer before and after its modification with Au nanoparticles, in the atmosphere of air, nitrogen, and 16 ppm of dimethyl sulfide. Dashed lines represent the equivalent model fitting.

**Figure 11:**
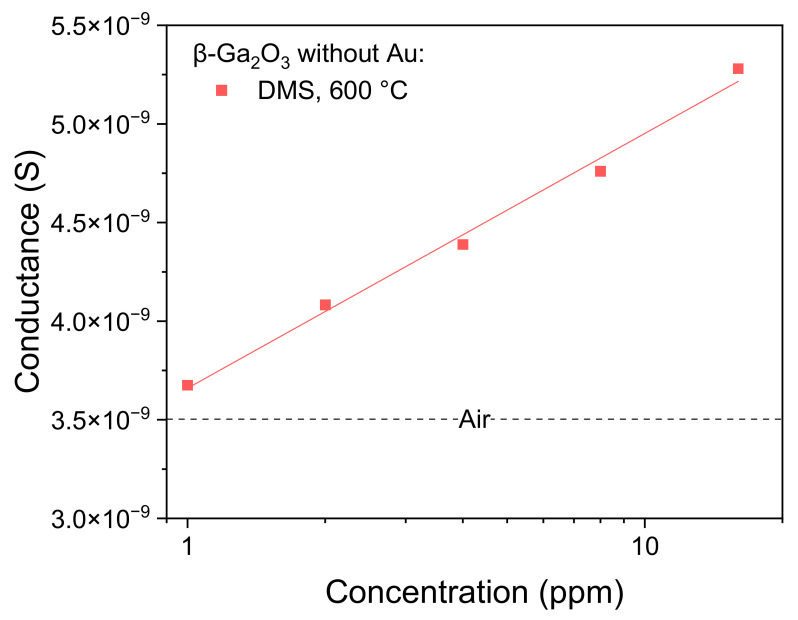
The conductance of the unmodified β-Ga_2_O_3_ epitaxial layer as a function of dimethyl sulfide concentration in the air (T ≈ 20 °C, RH ≈ 35%).

The equivalent circuit (Figure 9a) was fitted to the measured impedance spectra (Figure 10). The influence of 1–16 ppm of dimethyl sulfide (DMS) on the conductance of the layer *G*_layer_ = *1*/*R*_layer_ was tested for the unmodified β-Ga_2_O_3_ at 600 °C. The analysis of the results has shown that the conductivity of this metal oxide increases slightly with the concentration of DMS (Figure 11). Further experiments in the atmosphere containing dimethyl sulfide were performed only for a concertation of 16 ppm.

**Figure 12:**
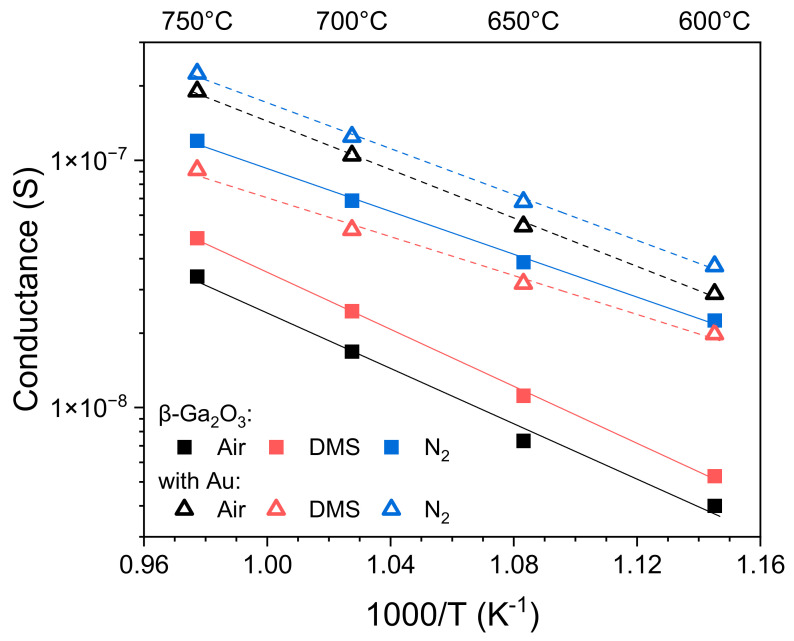
Conductance of β-Ga_2_O_3_ epitaxial layer, before and after its modification with Au nanoparticles, in the atmosphere of air, nitrogen, and air containing 16 ppm of dimethyl sulfide.

In air and in nitrogen, the conductance of the epitaxial layer increased with the temperature for both the unmodified and Au-modified sample. As predicted, the conductance of the unmodified layer was greater in nitrogen than in atmospheric air; however, in nitrogen, the conductance of the layer modified with gold nanoparticles increased very slightly. On the other hand, contrary to predictions and previous literature reports [33], the conductance of the layer increased after modification with Au nanoparticles (Figure 12).

The conductance of the unmodified β-Ga_2_O_3_ epitaxial layer increased slightly in the atmosphere containing dimethyl sulfide, compared to the ambient air (Figure 12). An opposite response was noticed for the layer modified with Au nanoparticles. Its conductance was significantly lower in the atmosphere containing dimethyl sulfide over the entire measured temperature range. In addition, the slope of the linear fit in the Arrhenius plot changed, which indicates a different nature of physicochemical processes occurring at the surface of the modified gallium oxide layer in the presence of dimethyl sulfide.

**Figure 13:**
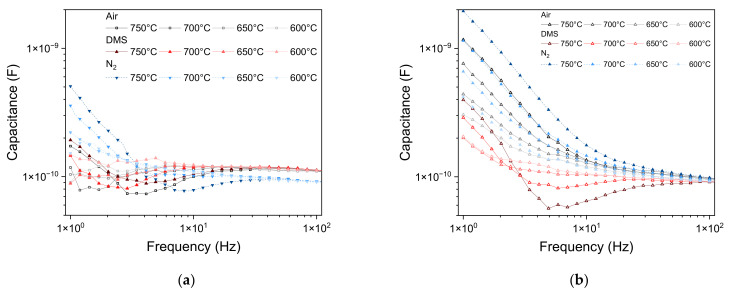
Low frequency capacitance of the parallel RC electric equivalent circuit of: (**a**) unmodified β-Ga_2_O_3_ epitaxial layer; (**b**) β-Ga_2_O_3_ epitaxial layer modified with Au nanoparticles. Data is smoothed with four-point adjacent-averaging.

For the unmodified β-Ga_2_O_3_ layer (in the atmosphere of air, nitrogen, DMS) (Figure 13a) and the layer modified with Au nanoparticles (in the atmosphere containing DMS) (Figure 13b), a decrease in capacitance in the 3–30 Hz frequency range is noticeable on the Bode diagram. This phenomenon, together with curling of the impedance spectra in the low frequency range (Figure 10), is likely caused by adsorption and desorption of chemical species at the surface of the metal oxide [42]. In the atmosphere containing DMS, these phenomena intensified with increasing temperature. Additionally, the drop in the capacitance was more pronounced for the epitaxial layer modified with Au nanoparticles. The noticeable decrease in the capacity for the unmodified layer measured in the ambient air at 750 °C may have been caused by the preceding series of measurements conducted in the atmosphere containing dimethyl sulfide (Figure 13a).

A sharp increase in capacitance was observed in the low frequency range (*f* < 3 Hz), which may have been caused by the accumulation of charges at the interface of metal oxide/gold electrodes and the formation of a depleted layer. The capacitance at low frequency increased with temperature, which can be explained by the decrease in the width of this depleted layer, which causes an increase in the capacity of the studied system [43]:

(5) $$C=\sqrt{\frac{e\varepsilon_{r}\varepsilon_{0}N_{d}}{2\phi }}=\frac{\varepsilon_{0}\varepsilon_{r}}{w},$$

where: *e*—electron charge, *ε*_0_—vacuum permittivity, *ε*_r_—relative permittivity, *N*_d_—donor concentration, *ϕ*—barrier height, and *w*—width of the depletion region.

Schipani et al. [43] suggest that an increase in layer capacitance in the low frequency range (*f* < 1 Hz) may be attributable to the charging and discharging of electron traps located in defects of the crystal structure at the boundaries outside of the depleted layers.

**Figure 14:**
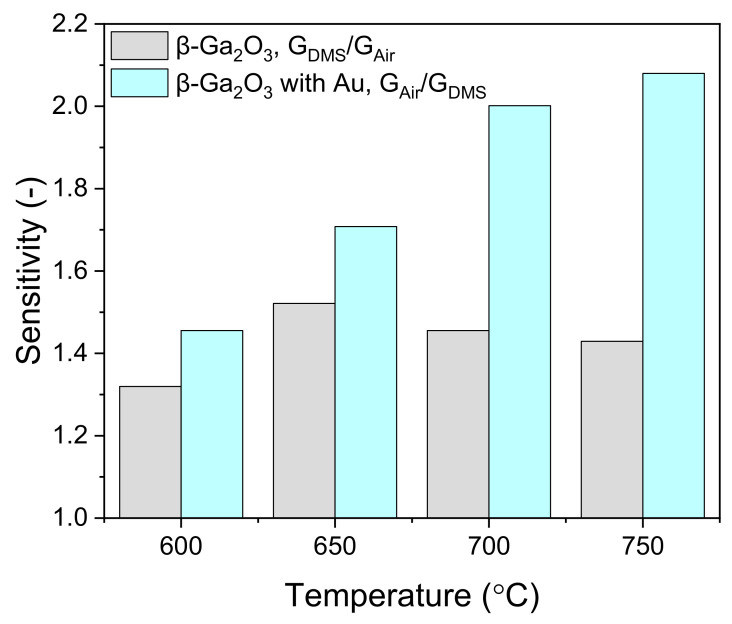
Sensitivity to 16 ppm of dimethyl sulfide of the unmodified and the modified β-Ga_2_O_3_ layer. The response of the β-Ga_2_O_3_ layer modified with Au nanoparticles is inversed; therefore, to keep the sensitivity greater than 1, the formula is also inversed (i.e., *S* = *G*_Air_/*G*_DMS_).

Since the conductance of the layer modified with Au nanoparticles decreased in the presence of dimethyl sulfide, its sensitivity (*S*) to DMS was defined as the ratio of conductance in the air to conductance in DMS, *S* = *G*_Air_/*G*_DMS_, analogous to how this parameter is defined in the case of chemoresistive sensors with a p-type sensor layer. The sensitivity of the unmodified β-Ga_2_O_3_ layer was low with a maximum at 650 °C; however, the sensitivity of the Au-modified layer increased with the temperature (Figure 14). This indicates a different nature of physicochemical process occurring at the surface of gallium oxide modified with gold nanoparticles.

## 4. Discussion

Ideally stoichiometric, undoped β-Ga_2_O_3_ is an insulator, while oxygen deficiency in the form of oxygen vacancies is responsible for conductivity at high temperature. Nonstoichiometric β-Ga_2_O_3_ is exclusively an n-type semiconductor at high temperature. The intrinsic n-type electrical conductivity of β-Ga_2_O_3_ has long been attributed to oxygen vacancies, as it has been for most metal oxides [15]. Changes in the conductivity of this material are attributed to changes in the concentration of free charge carriers, as a result of adsorption and desorption of oxygen ions at its surface and the diffusion of oxygen to the bulk of the crystal [44,45]. Thus, the conductivity of thin films of β-Ga_2_O_3_ depends on the concentration of oxygen in the ambient atmosphere [46].

It is well known that the conductivity *σ* of a porous polycrystalline semiconducting oxide depends on the conductivity of the grain boundary *σ*_gb_ and the conductivity of the bulk of the grains *σ*_b_ [47,48,49]:

(6) $$\sigma =\sigma_{\mathrm{gb}}+\sigma_{b}$$

(7) $$\sigma_{gb}=A\prime \cdot p_{\mathrm{gas}}^{n}\cdot \exp\left(\frac{eV_{S}}{kT}\right)$$

(8) $$\sigma_{b}=A\cdot \exp\left(\frac{E_{a}}{kT}\right),$$

where: *σ*_gb_—conductivity of the grain boundary, *σ*_gb_—conductivity of the grain bulk, *A*, *A′*—coefficients dependent on partial pressure of oxygen, *eV*_S_—height of the energy barrier between the grains, and *E*_a_—activation energy.

Both factors of the above function depend on the partial pressure of oxygen in the environment and on the temperature, but only the conductivity of the grain boundary *σ*_gb_ depends on the composition of the gas atmosphere. Thus, in the case of a constant concentration of oxygen, changes in conductance are caused by a change in temperature and are the result of physicochemical processes occurring at the surface and in the bulk of the sensor material. 

Four mechanisms of electrical conductivity are commonly distinguished: at the grain boundary, at the grain surface, in the grain bulk, on the Schottky contact [50]. Taking into account the microstructure of the studied gallium oxide (Figure 3), it can be assumed that the mechanism of electrical conductivity is determined by physicochemical processes occurring at the grain boundary. The results of electrical characterization of unmodified β-Ga_2_O_3_ confirm this assumption because its conductivity increases in the nitrogen atmosphere. In this atmosphere, oxygen adsorbed at the surface of the grains is desorbed, causing a change in the conductivity of the grain boundary. In addition, as indicated by literature data, bulk conductivity of grains also changes because the lower the partial pressure of oxygen, the more oxygen vacancies are formed [15,17]. However, the small change in conductance in the presence of dimethyl sulfide may result from too high operation temperature. It is difficult to identify the exact reactions that occur on the surface of the unmodified gallium oxide, as dimethyl sulfide can oxidize gradually:

(9) $${\mathrm{CH}}_{3}{\mathrm{SCH}}_{3}\overset{O_{2}}{\to }2{\mathrm{CH}}_{3}{\mathrm{SCH}}_{2}\mathrm{OO}\overset{T}{\to }{\mathrm{CH}}_{3}S\overset{O_{2}}{\to }{\mathrm{CH}}_{3}{\mathrm{SO}}_{2}\overset{T,O_{2}}{\to }{\mathrm{CO}}_{2}+{\mathrm{SO}}_{2}+H_{2}O$$

The response of β-Ga_2_O_3_ impregnated with Au nanoparticles is different. Its conductivity decreases in the atmosphere containing the reducing gas that is dimethyl sulfide. The activation energy determined on the basis of electrical measurements in air, nitrogen, and DMS atmosphere was clearly different for unmodified and modified β-Ga_2_O_3_ (Table 2). This indicates that the physicochemical processes taking place at the surfaces of these layers differ.

In the band theory of semiconductors, the phenomenon of bending of energy bands in the near-surface region is well known. There are many reasons for this, such as native surface states [51]. According to Stoneham [52], the bending of bands in oxide semiconductors reveals the general characteristics of the material in which anions are removed from the network nodes. Such a process occurs in semiconducting oxides with n-type conductivity. However, the main reason for the band bending at the surface of oxide materials properties characteristic of n-type semiconductors is the chemisorption of oxidizing gas (oxygen) or reducing gas (hydrogen), associated with electron exchange. Therefore, during the interaction of gases with the surface of n-type semiconductor materials, two main types of interactions are possible [53]. 

In the case of unmodified gallium oxide, taking into account the composition of the atmosphere in which the layers were studied, there was a bending of the bands at the grain boundary caused by the chemisorption of oxygen at the surface of the oxide and the exchange of electrons according to the reaction (10–12). It can be assumed that when establishing the equilibrium of the adsorption process, only the simplest intermediate stages occur:

(10) $$O_{2}+e^{-}⇄O_{2}^{-}$$

(11) $$O_{2}⇄2O$$

(12) $$O+e^{-}⇄O^{-}$$

At low oxygen pressure and/or high temperature, the dominant ionic form of oxygen at the surface will be $O^{-}$ ions.

In the case of β-Ga_2_O_3_ impregnated with Au nanoparticles, both the properties of gallium oxide and gold should be taken into account. Analyzing the catalytic properties of gold particles deposited on metal oxides with semiconductor properties, it is widely believed that the dominant role is played by oxygen molecules that dissociate at the surface of gold [54,55]. At elevated temperatures, atomic oxygen recombines to O_2_. The recombination temperature depends on the crystallographic orientation of the surface of gold. It occurs at: 377 °C on polycrystalline gold [56], 317 °C at the surface with (110) orientation [57], and as low as 200 °C at the surface with (100) orientation [58]. Apart from the chemical aspect, the electronic aspect must also be taken into account [59,60]

Au nanoparticles randomly scattered on the surface of β-Ga_2_O_3_ grains create Schottky barriers between metallic Au island and semiconductor β-Ga_2_O_3_ grains. The work function of gallium oxide n-type semiconductor is 4.1 eV, which is much smaller than that of gold, 5.1 eV. Since the Fermi levels of Au and β-Ga_2_O_3_ must equalize, the electrons migrate from β-Ga_2_O_3_ to Au, and therefore the Schottky barrier is formed at the β-Ga_2_O_3_/Au interface [60]. Thus, gold nanoparticles cause local changes in the Fermi level of gallium oxide [61]. As a result, the Au islands charge negatively, and in their vicinity a charge depleted layer forms on the surface of the β-Ga_2_O_3_ grains. 

**Figure 15:**
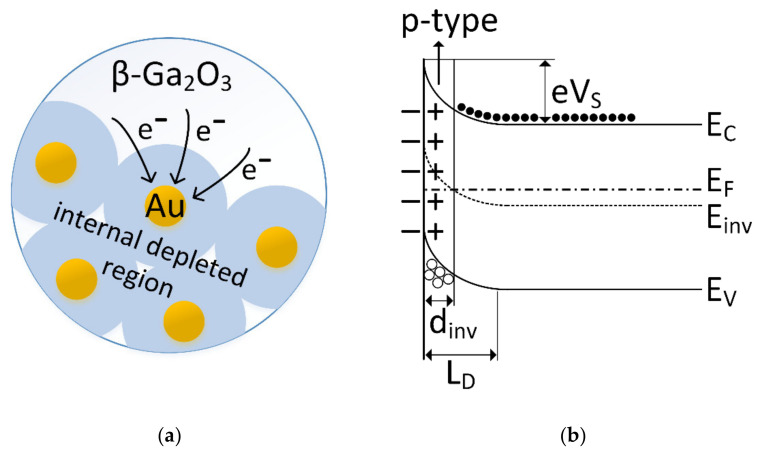
(**a**) Internal depleted layer at the β-Ga_2_O_3_/Au phase boundary; (**b**) corresponding band diagram; E_inv_—energy of the inversion level, E_F_—energy of the Fermi level, E_C_—energy of the lower edge of the conduction band, E_V_—energy of the upper edge of the valence band, L_D_—width of the depleted layer, d_inv_—width of the inversion layer, ○—holes, ●—electrons, *e*V_S_—height of the potential barrier at the surface of the grain.

For this reason, the conductance of β-Ga_2_O_3_ modified with gold nanoparticles should be lower than that of pure β-Ga_2_O_3_. However, the impregnation with Au nanoparticles of a thin layer of gallium oxide made of grains with a much larger diameter than the Au nanoparticles resulted in an increase in the conductivity of the layer and anomalous response in the presence of dimethyl sulfide. The reason for this may be the phenomenon of inversion on the surface of gas-sensitive material [62,63] (Figure 15), where holes become the charge carriers (p-type semiconductivity).

An analysis of the results of this study leads us to the conclusion that the Au/β-Ga_2_O_3_ contact is responsible for the unusual electrical response. The height of the potential barrier formed at the β-Ga_2_O_3_ grain and Au boundary depends on the difference in work functions *ϕ*_β-Ga_2_O_3__ = 4.1 eV [64] and *ϕ*_Au_ = 5.1 eV [65], hence the built-in potential:

(13) $$V_{bi}=\frac{\phi_{Au}-\phi_{\beta -Ga2O3}}{q}≅1\;\mathrm{eV}$$

**Figure 16:**
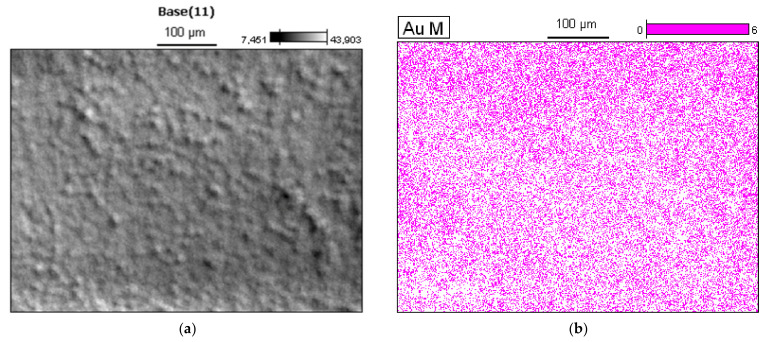
(**a**) SEM/EDS image of the surface of the β-Ga_2_O_3_ layer; (**b**) distribution map of Au nanoparticles on the surface of the β-Ga_2_O_3_ layer. Determined by the EDS method.

The width of the depletion layer depends also on the electrical permeability of the semiconductor and the concentration of donors. The relative permeability of β-Ga_2_O_3_ is 9.9–10.2 [66] or 13.5 [67]. It is assumed that the concentration of donors *N*_D_ in gallium oxide can range from 6 × 10^22^ to 8 × 10^23^ m^−3^ [68]. Using the equation [51]:

(14) $$w=\sqrt{\frac{2\varepsilon }{qN_{D}}\left(V_{\mathrm{bi}}-V-\frac{kT}{q}\right)},$$

where: ε—semiconductor permittivity, *N*_D_—donor density, *V*_bi_—built-in potential, *V*—voltage, *q*—electronic charge, *T*—temperature, and assuming that *V* and *kT/q* are much smaller than *V*_bi_ it can be estimated that for *N*_D_ = 6 × 10^22^ m^−3^ the width of the depleted layer is approximately 35 nm. Thus, if the distance between the gold nanoparticles is at least 70 nm, then the entire surface of gallium oxide will be covered with a depleted layer and inversion should occur (Figure 16). 

If dimethyl sulfide is present in the atmosphere, where molecules show a high affinity for gold, then they will form a covalent bond together with Au nanoparticles [69]. As a result, the thickness of the inversion layer will decrease because some of the electrons returning to gallium oxide will recombine with the holes of the inversion layer. The conductance of the β-Ga_2_O_3_ layer modified with Au nanoparticles will decrease as a result of these physicochemical processes.

## 5. Conclusions

In this work, a β-Ga_2_O_3_ epitaxial layer surface-modified with Au nanoparticles was investigated. The results of the electrical characterization of the epitaxial layer before modification in atmospheric air, dry nitrogen, and air containing dimethyl sulfide were typical to an n-type semiconductor sensor material. However, the conductance of the β-Ga_2_O_3_ epitaxial layer after modification with Au nanoparticles changed in an unusual way. The conductance of the layer did not decrease but, instead, it increased after modification with Au nanoparticles. The conductance in the atmosphere of air and nitrogen was similar. Only a slight increase of the conductance was observed in the atmosphere of nitrogen. In the atmosphere of air containing dimethyl sulfide the conductance decreased, which is characteristic of the p-type sensor layers.

The increase of conductance of the β-Ga_2_O_3_ epitaxial layer after modification with Au nanoparticles may be the result of the formation of an inversion layer on the surface of the metal oxide grains and a change in the type of conductivity from the n-type to the p-type. In other words, the surface of β-Ga_2_O_3_, which is an n-type semiconductor, after modification with Au nanoparticles was depleted of donor carriers (electrons) to such an extent that the holes became the majority carriers on the surface. According to the authors, this is consistent with previous studies, in which the conductance of β-Ga_2_O_3_ decreased after the introduction of gold particles [33]; however, in the case of the layer described in this paper, the concentration of gold nanoparticles at the surface of the grains was sufficient for the occurrence of the phenomenon of inversion of the conductivity type. In addition, the occurrence of p-type inversion is also evidenced by the anomalous electrical response of the β-Ga_2_O_3_ layer in the presence of dimethyl sulfide. The modification of the material resulted in an increase in sensitivity to this compound; however, it still remained low compared to the gas sensitive layers based on metal oxide nanomaterials described in the literature [70]. This demonstrates the superiority of nanomaterials with an extremely large active surface area that affects changes in sensitivity. Due to the difficulty in p-type doping of β-Ga_2_O_3_, the phenomenon of inversion, observed as a result of modification of the surface of this material with gold nanoparticles, should be studied more closely.

## Figures and Tables

**Table 1:** Oxygen bonds’ atomic concentration in O1s region.

Oxygen Bonds	Unmodified β-Ga_2_O_3_	β-Ga_2_O_3_ Impregnated with Au Nanoparticles
Ga-O	5.68%	4.61%
O-C, O=C	19.30%	17.48%
OH^-^	1.94%	4.54%

**Table 2:** The value of the activation energy *E*_a_ of β-Ga_2_O_3_ layers in various atmospheres.

Atmosphere Composition	Unmodified β-Ga_2_O_3_	β-Ga_2_O_3_ Impregnated with Au Nanoparticles
Ambient air	(1.11 ± 0.07) eV	(0.97 ± 0.03) eV
Dry nitrogen	(0.86 ± 0.03) eV	(0.92 ± 0.03) eV
Ambient air + 16 ppm dimethyl sulfide	(1.14 ± 0.03) eV	(0.78 ± 0.05) eV

## Data Availability

The data that support the findings of this study are available from the corresponding author upon reasonable request.
